# Multifaceted stress response in Nile tilapia (*Oreochromis niloticus*) fingerlings: integrative analysis of salinity, ammonia, and stocking density effects on growth, physiology, and gene expression

**DOI:** 10.1007/s10695-025-01462-6

**Published:** 2025-02-13

**Authors:** Sameh Metwaly, Hala Nasr, Khalifa Ahmed, Mohamed Fathi

**Affiliations:** 1 WorldFish, Abbassa, Abou Hammad, Egypt; 2 Department of Fish Health Management, Central Laboratory for Aquaculture Research, Agriculture Research Center, Abbassa, Abou Hammad, Sharkia Egypt; 3 Kafr El-Sheikh University, Kafr El-Sheikh, Egypt; 4 Professor and of Biochemistry, Faculty of Veterinary Medicine, Zagazig University, Zagazig, Egypt; 5 National Institute of Oceanography and Fisheries (NIOF), Cairo, Egypt

**Keywords:** Tilapia, Salinity stress, Ammonia toxicity, Stocking density, Osmoregulation, Growth performance, Gene expression

## Abstract

This study investigated the effects of salinity, ammonia, and stocking density on Nile tilapia (*Oreochromis niloticus*) fingerlings over a 74-days. In three separate experiments, fingerlings (initial weight 25 ± 2.4 g) were exposed to salinity levels (5, 10, 15, and 20 ppt), ammonia concentrations (0.01, 0.02, 0.1, and 0.2 mg/L), and stocking densities (10, 15, 20, and 25 fish per 96 L aquarium). Survival, growth performance, biochemical parameters, and gene expression changes were assessed. Salinity ≥ 15 ppt and ammonia ≥ 0.1 mg/L significantly impaired growth (final weight, weight gain, specific growth rate, and feed efficiency) and increased mortality rates, reaching 37% and 56% at 20 ppt salinity and 0.2 mg/L ammonia, respectively. Elevated salinity and ammonia also caused significant increases in the activities of ALT, AST, LDH enzymes, along with higher serum glucose levels, while disrupting serum protein and ion concentrations, indicating considerable metabolic and osmoregulatory disturbances. At the molecular level, the expression of the growth-promoting IGF-I gene was down-regulated, while inflammatory marker TNFα was up-regulated, suggesting compromised health. Stocking density had less pronounced effects, though densities ≥ 20 fish/aquarium led to reduced growth, altered biochemical markers, and gene expression changes compared to 10–15 fish/aquarium. These findings establish salinity and ammonia tolerance thresholds for tilapia fingerlings, emphasize optimal stocking density, and provide insights into the physiological and molecular responses to multifactorial stressors. The study contributes to sustainable management strategies for tilapia aquaculture under variable environmental conditions.

## Introduction

Aquaculture plays a vital role in global food production, meeting the increasing demand for protein as wild fisheries approach their exploitation limits. It is the fastest-growing sector in animal-based food production, currently supplying nearly half of the world’s fish consumption (FAO 2020). Among the species cultivated, Nile tilapia (Oreochromis niloticus) is a cornerstone of the industry due to its rapid growth, adaptability, and broad consumer appeal.

The success of Nile tilapia in aquaculture is largely attributed to its omnivorous diet, wide environmental tolerance, and ease of breeding in captivity (El-Sayed 2006). Innovations such as the development of Genetically Improved Farmed Tilapia (GIFT) strains have improved growth performance and disease resistance (Komen and Troutman 2019). Similarly, advances in alternative protein sources aim to reduce dependence on fishmeal and enhance the sustainability of tilapia farming systems (Tacon et al. 2022 ). Despite these advancements, significant challenges remain in optimizing environmental conditions, particularly regarding salinity fluctuations, ammonia toxicity, and stocking density.

Salinity is a critical environmental factor influencing tilapia physiology and growth. Although Nile tilapia is a freshwater species, its euryhaline nature enables survival in brackish waters. However, salinity levels exceeding optimal thresholds can disrupt osmoregulation, metabolic homeostasis, and growth (El-Sayed 2006; Rahmah et al. 2020). The physiological mechanisms supporting salinity adaptation, including the roles of cortisol, growth hormone (GH), and insulin-like growth factor 1 (IGF-1), have been studied (McCormick 2001; Sakamoto and McCormick 2006), yet the precise salinity tolerance limits for Nile tilapia fingerlings require further clarification.

Ammonia toxicity is another major concern in intensive aquaculture systems. Ammonia, a byproduct of protein metabolism and organic matter decomposition, can accumulate to harmful levels, especially under suboptimal water management. Chronic exposure to elevated ammonia levels impairs growth, immune function, and survival (Randall and Tsui 2002). It can induce oxidative stress, disrupt antioxidant defenses, and lead to tissue damage, as evidenced by elevated circulating glucose, cortisol, aspartate aminotransferase (AST), and alanine aminotransferase (ALT) levels (Zhang et al. 2018; Yousefi et al. 2021).

Stocking density is another critical factor in aquaculture systems, directly influencing water quality, feed efficiency, growth rates, and overall fish welfare. High stocking densities can exacerbate stress, impair physiological functions, and reduce feed efficiency, while excessively low densities may disturb social behaviors such as shoaling (El-Sayed and Kawanna 2008; Chambel et al. 2015). Optimizing stocking density is therefore essential to balance growth performance and welfare outcomes.

Despite numerous studies investigating the individual effects of salinity, ammonia, and stocking density, the combined impacts of these stressors on tilapia fingerlings remain poorly understood. Tilapia fingerlings are a sensitive life stage, and their health and growth during this phase are critical to overall production success. The interactions between salinity, ammonia, and stocking density may compound stress responses, resulting in metabolic dysfunction and impaired growth.

This study aims to fill this knowledge gap by comprehensively evaluating the effects of salinity, ammonia concentration, and stocking density on Nile tilapia fingerlings. Through integrative physiological, biochemical, and molecular analyses, this research defines tolerance thresholds for these environmental factors, provides insights into the underlying mechanisms of stress adaptation, and contributes to the development of sustainable management strategies for tilapia aquaculture under variable environmental conditions.

## Material and methods

### Experimental fish and husbandry

Mono-sex Nile tilapia (*Oreochromus niloticus*) fingerlings in apparent good health were sourced from the nursery ponds of the Central Laboratory for Aquaculture Research, Abbassa, Abo-Hammad, Sharqia, Egypt. Prior to the experiment, the fingerlings were acclimatized in indoor fiberglass tanks for two weeks. A total of 450 fingerlings with an initial mean weight of 25 ± 2.4 g were used for the study.

### Experimental design

To evaluate the effects of salinity, ammonia, and stocking density on Nile tilapia (*Oreochromis niloticus*) fingerlings, three independent experiments were conducted over a 74-day period. The experimental setup utilized 36 glass aquaria, each with dimensions of 80 × 40 × 30 cm and a water-holding capacity of 96 L. Each aquarium was equipped with an air stone for continuous aeration and a thermostatically controlled heater to maintain stable water temperature. A summary of the treatments and experimental design is provided in Table 1.

**Table 1:** Experimental design to assess the effects of salinity, ammonia, and stocking density on Nile tilapia fingerlings

	T1 (control)	T2	T3	T4
Experiment 1 (Salinity)	5 ppt, 30 fish	10 ppt, 30 fish	15 ppt, 30 fish	20 ppt, 30 fish
Experiment 2 (ammonia)	0.01 mg/L, 30 fish	0.02 mg/L, 30 fish	0.1 mg/L, 30 fish	0.2 mg/L, 30 fish
Experiment 3 (Stocking Density)	30 fish (10 fish/ aquarium)	45 fish (15 fish/ aquarium)	60 fish (20 fish/ aquarium)	75 fish (25 fish/ aquarium)

### Salinity experiment

The salinity experiment included four treatment levels: 5 ppt (control), 10 ppt, 15 ppt, and 20 ppt. Salinity adjustments were made by dissolving pre-measured amounts of marine salt (Instant Ocean®) in dechlorinated tap water. A YSI Pro30 Conductivity Meter was used to monitor salinity levels daily, ensuring precise and consistent salinity in each treatment group. To maintain stable water quality, 50% of the water in each aquarium was replaced daily with freshly prepared water at the same salinity level. This approach ensured uniformity and minimized variability in salinity across replicates.

### Ammonia experiment

The ammonia experiment tested four concentrations of total ammonia nitrogen (TAN): 0.01 mg/L (control), 0.02 mg/L, 0.1 mg/L, and 0.2 mg/L. Analytical-grade ammonium chloride (NH₄Cl) was used to prepare stock solutions, which were subsequently added to the aquaria to achieve the desired ammonia concentrations. TAN levels were monitored daily using a calibrated API Ammonia Test Kit to ensure that the ammonia concentrations remained within the target range. Any deviations were corrected by adjusting the NH₄Cl input. Daily water replacement (50% of total volume) was conducted using pre-prepared water containing the exact ammonia concentration, ensuring uniform and consistent TAN levels across all replicates.

### Stocking density experiment

The stocking density experiment investigated the effects of fish density on growth and health. Four stocking densities were used: 10 fish/aquarium (low density), 15 fish/aquarium, 20 fish/aquarium, and 25 fish/aquarium (high density). The initial stocking weights of the fish were standardized to minimize variability. Each aquarium was managed to maintain consistent feeding, aeration, and water quality conditions. Daily monitoring of water quality parameters, including dissolved oxygen, temperature, pH, nitrite, nitrate, and TAN, ensured uniformity across treatments.

### Water quality management

The aquaria were filled with dechlorinated tap water, and 50% of the water volume was replaced daily to remove waste and maintain optimal conditions. Key water quality parameters were monitored daily using calibrated instruments: dissolved oxygen levels were maintained at 6–7 mg/L (YSI Pro20 Dissolved Oxygen Meter), temperature at 26 ± 2°C (YSI Pro20 Thermometer), and pH at 7.5 (Hanna HI98190 pH Meter). Nitrite and nitrate concentrations were kept at 0.05 mg/L and 10 mg/L, respectively, using API test kits, while ammonium levels were maintained at 0.01 mg/L (API Ammonia Test Kit). In salinity experiments, salinity levels were measured and adjusted using a YSI Pro30 Conductivity Meter.

### Feeding regime

The fish were fed a commercial diet containing 32% crude protein, 5% lipid (Aller Aqua, Egypt) three times daily at 09:00, 12:00, and 15:00 h. The feeding rate was 3% of body weight, adjusted biweekly based on fish biomass.

### Fish sampling and measurements

Every two weeks, fish from each aquarium were counted and group-weighed to the nearest 0.1 g. Feed amounts were adjusted based on updated biomass **(**Reda 2009**)**. The experiments were conducted for 74-days.

### Health assessment

Daily observations were made to monitor mortality and behavioral changes. At the end of each experiment, liver samples were collected from four fish per treatment for histological examination.

### Growth performance and feed efficiency

Growth performance and feed efficiency were evaluated using standard formulas: weight gain (WG%) was calculated as [(Average final weight—Average initial weight) / Average initial weight] × 100, following Abdelhamid et al. (2019); specific growth rate (SGR%) was determined as 100 × [ln(final body weight)—ln(initial body weight)] / experimental period (days), as described by Hopkins (1992); and feed conversion ratio (FCR) was calculated as the total feed consumed (g) divided by the total weight gain (g).

### Biochemical analysis

Blood samples were collected from the caudal vein following the method of Feldman et al. (2000) and centrifuged as described by Aly et al. (2008) to obtain serum for biochemical analysis. The analyzed parameters included aspartate aminotransferase (AST) and alanine aminotransferase (ALT) activities (Tietz 1976), total protein, albumin, and globulin concentrations (Grant et al. 1987; Doumas et al. 1981; Doumas and Biggs 1972), lactate dehydrogenase (LDH) activity (Wacker 1956; Gay et al. 1968), serum glucose levels (Trinder 1969), and serum sodium and potassium levels (Henry et al. 1974; Luppa et al. 2001).

## Molecular analysis

### RNA extraction and cDNA synthesis

Total RNA was extracted from head kidney tissue using the RNeasy Mini Kit (Qiagen, Germany). RNA quality and concentration were determined using spectrophotometrically (Wilfinger et al. 1997). cDNA synthesis was performed using the Quantitect® Reverse Transcription Kit (Qiagen, Germany).

### Quantitative RT-PCR

Gene expression of insulin-like growth factor-1 (IGF1) and tumor necrosis factor-alpha (TNF-α) was conducted using the primers listed in Table 2. EF-1α served as the reference gene. PCR conditions were: 94 °C for 5 min, followed by 40 cycles of 94 °C for 10 s, 55 °C for 30 s, and 72 °C for 40 s, with a final extension at 72 °C for 7 min. Melt curve analysis was performed using Applied Biosystem real-time PCR system software.

**Table 2:** Primers used for the examination of the expressions of cytokine genes in the head kidney tissues of *Oreochromis niloticus*

Gene	Primer sequence (5’−3’)	Reference
EF-1α	CCTTCAACGCTCAGGTCATC	Gröner et al. 2015
	TGTGGGCAGTGTGGCAATC	
IGF1	TTCTCCAAAAACGAGCCTGCG	Vera cruz et al. 2006
	TCTGCTACTAACCTTGGGTGC	
*TNF α*	CCAGAAGCACTAAAGGCGAAGA	Standen et al. 2016
	CCTTGGCTTTGCTGCTGATC	

Data Analysis: Gene expression data were analyzed using the 2^-ΔΔCt method (Yuan et al. 2006).

### Statistical analysis

Data were subjected to one-way ANOVA using SPSS (version 21.0). Homogeneity of variance was tested, and Duncan’s test was used for post-hoc comparisons. Differences were considered significant at *P* < 0.05.

## Results

The study assessed the impact of salinity, ammonia concentration, and stocking density on Nile tilapia (*Oreochromis niloticus*) fingerlings over a 12-week period, focusing on survival, growth performance, biochemical parameters, and molecular responses.

Survival rates were significantly affected by environmental stressors. In the salinity experiment, there was no mortality at 5 ppt and 10 ppt, but mortality increased to 16.67% at 15 ppt and 33.33% at 20 ppt (Table 3). Fish subjected to 20 ppt showed clear signs of stress, such as darkening of color and tendency to remain near the bottom (bottom-dwelling behavior). In the ammonia experiment, mortality was dose-dependent, ranging from 3.33% in the control group (0.01 mg/L) to 46.67% at 0.2 mg/L ammonia (Table 4). The impact of stocking density on survival was less pronounced, no mortality occurred at 10 and 15 fish per aquarium, but mortality reaching 13.33% and 23.33% mortality at 20 and 25 fish per aquarium, respectively (Table 5).

**Table 3:** Effect of salinity level on survival of *Oreochromis niloticus* over the experimental period

Group No	Salinity	No. of Tilapia fish	Total mortality	
			No	%
Control	5 ppt	30	0	0.0
2	10 ppt	30	0	0.0
3	15 ppt	30	5	16.67
4	20 ppt	30	10	33.33

**Table 4:** Effect of ammonia concentration on survival of *Oreochromis niloticus* over the experimental period

Group No	Ammonia	No. of Tilapia fish	Total mortality	
			No	%
Control	0.01	30	1	3.33
2	0.02	30	3	10
3	0.10	30	8	26.67
4	0.20	30	14	46.67

**Table 5:** Effect of stocking density on survival of *Oreochromis niloticus* over the experimental period

Group No	Stocking density	No. of Tilapia fish	Total mortality	
			No	%
Control	10	30	0	0
2	15	45	0	0
3	20	60	4	13.33
4	25	75	7	23.33

Growth performance, assessed via final weight (FW), body gain (BG), and specific growth rate (SGR), showed significant declines as salinity and ammonia concentration increased. The largest reductions were observed at 20 ppt salinity and 0.2 mg/L ammonia. Interestingly, the stocking density experiment, the optimal growth performance occurred at 15 fish per aquarium, while the lowest growth was recorded at 25 fish per aquarium, suggesting a density-dependent effect on growth (Tables 6, 7 & 8).

**Table 6:** Effect of salinity on growth performance and feed efficiency

Parameter	Control	10 ppt	15 ppt	20 ppt
IW (gm)	25.11 ± 0.09	24.95 ± 0.07	25.07 ± 0.10	24.96 ± 0.09
FW (gm)	45.02 ± 0.18^a^	41.24 ± 0.21^b^	38.85 ± 0.39^c^	34.79 ± 0.60^d^
BG %	79.33 ± 0.23^a^	65.28 ± 1.04^b^	54.99 ± 2.1^c^	39.36 ± 2.05^d^
SGR %	0.97 ± 0.00^a^	0.84 ± 0.01^b^	0.73 ± 0.02^c^	0.55 ± 0.02^d^

**Table 7:** Effect of ammonia on growth performance and feed efficiency

Parameter	Control	0.02mg/dL	0.1mg/dL	0.2mg/dl
IW (gm)	25.11 ± 0.09	24.98 ± 0.01	25.30 ± 0.14	25.06 ± 0.05
FW (gm)	45.02 ± 0.18^a^	43.17 ± 0.53^b^	36.94 ± 0.45^c^	23.96 ± 0.25^d^
BG %	79.33 ± 0.23^a^	72.80 ± 2.05^b^	46.02 ± 1.77^c^	31.54 ± 1.23^c^
SGR %	0.97 ± 0.00^a^	0.91 ± 0.02^b^	0.63 ± 0.02^c^	0.46 ± 0.02^d^

**Table 8:** Effect of stocking density on growth performance and feed efficiency

Parameter	Control (10 Fish/aquaria)	15 (Fish/aquaria)	20 (Fish/ aquaria)	25 (Fish/ aquaria)
IW (gm)	25.11 ± 0.09	25.16 ± 0.04	25.09 ± 0.10	25.05 ± 0.01
FW (gm)	45.02 ± 0.18^a^	42.45 ± 0.42^b^	39.08 ± 0.43^c^	36.73 ± 0.40^d^
BG %	79.33 ± 0.23^a^	68.71 ± 1.55^b^	55.79 ± 2.14^c^	46.62 ± 1.68^d^
SGR %	0.97 ± 0.00^a^	0.87 ± 0.02^b^	0.74 ± 0.02^c^	0.64 ± 0.02^d^

Biochemical analysis across all experiments demonstrated consistent trends. Increased environmental stressor significant elevated the levels of aspartate aminotransferase (AST), alanine aminotransferase (ALT), and lactate dehydrogenase (LDH). While serum total protein, albumin, and globulins levels decreased. Serum glucose levels rose with increasing the stress, especially at the highest concentrations of salinity, ammonia and stocking density. Disrupted osmoregulation was evident, with serum sodium levels decreasing and potassium levels increasing as stress intensified (Tables 9, 10  and 11).

**Table 9:** Effect of salinity on some blood parameters

Parameter	Control	10 ppt	15 ppt	20 ppt
ALT (u/l)	23.00 ± 0.96^b^	24.11 ± 1.35^b^	26.00 ± 1.76^b^	33.42 ± 0.90^a^
AST (u/l)	40.40 ± 032^d^	43.17 ± 0.42^c^	45.80 ± 0.95^b^	57.20 ± 0.51^a^
LDH (u/l)	489.97 ± 8.14^c^	504.98 ± 1.58^c^	526.91 ± 7.70^b^	563.75 ± 1.85^a^
Total protein (g/dL)	5.66 ± 0.09^a^	5.18 ± 0.03^b^	5.02 ± 0.05^b^	4.38 ± 0.18^c^
Albumin (g/dL)	3.75 ± 0.12^a^	2.86 ± 1.07^b^	2.27 ± 0.12^c^	2.17 ± 0.01^c^
Globulins (g/dL)	2.85 ± 0.06^a^	2.33 ± 0.04^b^	2.11 ± 0.23^b^	1.91 ± 0.21^b^
Glucose (mg/dL)	90.20 ± 1.28^b^	98.60 ± 0.96^a^	99.30 ± 0.67^a^	103.33 ± 2.68^a^
Na^+^ (mEq/L)	193.30 ± 0.74^a^	181.40 ± 0.88^b^	177.40 ± 1.40^b^	143.80 ± 2.38^c^
K^+^ (mEq/L)	4.67 ± 0.33^c^	5.76 ± 0.32^b^	7.88 ± 0.36^b^	10.41 ± 0.32^a^

**Table 10:** Effect of ammonia on some blood parameters

Parameter	Control	0.02mg/dL	0.1mg/dL	0.2mg/dL
ALT (u/l)	23.00 ± 0.96^b^	24.62 ± 1.21^b^	25.60 ± 0.58^b^	32.73 ± 0.93^a^
AST (u/l)	40.44 ± 0.32^c^	41.19 ± 0.70^c^	43.80 ± 0.45^b^	51.54 ± 0.58^a^
LDH (u/l)	489.97 ± 8.14^c^	700.87 ± 3.79^b^	760.69 ± 24.28^ab^	791.30 ± 26.52^a^
Total protein (g/dL)	5.66 ± 0.09^a^	5.35 ± 0.08^a^	4.48 ± 0.23^b^	4.07 ± 0.04^b^
Albumin (g/dL)	3.82 ± 0.06^a^	3.75 ± 0.12^a^	2.78 ± 0.16^b^	2.78 ± 0.06^b^
Globulin (g/dL)	2.76 ± 0.03^a^	2.28 ± 0.03^b^	2.12 ± 0.31^b^	1.88 ± 0.22^b^
Glucose (mg/dL)	92.44 ± 3.03^c^	95.77 ± 1.21^bc^	99.30 ± 0.67^ab^	102.63 ± 0.44^a^
Na^+^ (mEq/L)	197.43 ± 0.96^a^	177.46 ± 1.47^b^	177.02 ± 0.45^b^	167.13 ± 0.90^c^
K^+^ (mEq/L)	4.67 ± 0.33^d^	7.33 ± 0.03^c^	9.09 ± 0.11^b^	10.80 ± 0.14^a^

**Table 11:** Effect of stocking density of *Oreochromis niloticus* on some blood parameters

Parameter	Control (10 Fish/aquaria)	15 (Fish/aquaria)	20 (Fish/ aquaria)	25 (Fish/ aquaria)
ALT (u/L)	23.00 ± 0.96^b^	24.99 ± 0.35^b^	25.45 ± 1.21^b^	34.61 ± 0.53^a^
AST (u/L)	40.40 ± 0.32^b^	42.18 ± 1.05^b^	42.32 ± 0.74^b^	47.63 ± 0.92^a^
LDH (u/L)	480.06 ± 2.12^b^	489.97 ± 8.14^b^	503.90 ± 2.85^b^	564.83 ± 12.38^a^
Total protein (g/dL)	5.62 ± 0.09^a^	5.51 ± 0.09^a^	5.16 ± 0.09^a^	4.48 ± 0.23^b^
Albumin (g/dL)	3.81 ± 0.03^a^	3.75 ± 0.12^a^	3.60 ± 0.09^a^	2.66 ± 0.04^b^
Globulin (g/dL)	2.30 ± 0.04^a^	1.91 ± 0.21^ab^	1.87 ± 0.14^ab^	1.81 ± 0.11^b^
Glucose (mg/dL)	97.77 ± 1.38^b^	99.30 ± 0.67^b^	103.34 ± 1.52^a^	107.29 ± 1.18^a^
Na^+^ mEq/L	183.12 ± 0.93^a^	177.48 ± 1.47^b^	173.15 ± 0.75^c^	142.15 ± 0.65^d^
K^+^ mEq/L	3.86 ± 0.31^c^	4.67 ± 0.23^c^	5.70 ± 0.32^b^	6.86 ± 0.33^a^

Gene expression analysis revealed consistent down-regulation of insulin-like growth factor I (IGF-I) and an up-regulation of tumor necrosis factor alpha (TNFα) in response to increased environmental stress. In the salinity experiment, IGF-I expression was highest at 10 ppt and lowest at 20 ppt, while TNFα showed the opposite trend. Similar patterns were observed in the ammonia and stocking density experiments, with the most significant gene expression changes observed at the highest stress levels:0.2 mg/L ammonia and 25 fish per aquarium, respectively (Tables 12, 13 and 14).

**Table 12:** Effect of salinity on IGF-I and TNFα

Parameter	Control	10 ppt	15 ppt	20 ppt
IGF-I	1.00 ± 0.00^a^	0.82 ± 0.01^b^	0.53 ± 0.04^c^	0.32 ± 0.03^d^
TNFα	1.00 ± 0.00^c^	1.39 ± 0.02^c^	5.20 ± 0.63^b^	10.63 ± 0.70^a^

**Table 13:** Effect of ammonia on IGF-I and TNFα

Parameter	Control	0.02mg/dL	0.1mg/dL	0.2mg/dL
IGF-I	1.00 ± 0.00^a^	0.57 ± 0.04^b^	0.29 ± 0.04^c^	0.06 ± 0.01^d^
TNFα	1.00 ± 0.00^d^	6.90 ± 0.08^c^	10.65 ± 0.70^b^	15.14 ± 0.12^a^

**Table 14:** Effect of stocking density on IGF-I and TNFα

Parameter	Control (10 Fish/aquaria)	15 (Fish/aquaria)	20 (Fish/ aquaria)	25 (Fish/ aquaria)
IGF-I	1.00 ± 0.00^a^	0.20 ± 0.00^b^	0.09 ± 0.01^c^	0.03 ± 0.00^d^
TNFα	1.00 ± 0.00^d^	6.94 ± 0.51^c^	10.93 ± 0.13^b^	13.15 ± 0.18^a^

Values are presented as mean ± standard error (SE) of 5 replicates per treatment. Means within the same row followed by different superscript letters are significantly different (*p* < 0.05) as determined by Duncan’s multiple range test. Superscript letters are assigned alphabetically, with ’a’ representing the highest mean value and subsequent letters indicating progressively lower values.

## Discussion

This study comprehensively evaluated the effects of salinity, ammonia nitrogen concentration, and stocking density on the survival, growth performance, biochemical parameters, and gene expression of Nile tilapia (*Oreochromis niloticus*) fingerlings. The findings elucidate the complex interplay between these environmental stressors and fish physiology, providing valuable insights into optimizing aquaculture practices for sustainable production.

### Effects of salinity

The results indicate a significant decline in survival and growth performance at salinity levels above 10 ppt, with adverse physiological responses observed as salinity increased. These findings align with previous studies (El-Sayed 2006; Lawson and Anetekhai, 2011), which reported reduced tolerance thresholds in Nile tilapia under high salinity. Elevated salinity disrupted osmoregulatory functions, as evidenced by significant shifts in serum ion levels and protein profiles. Elevated enzyme activities, including aspartate aminotransferase (AST), alanine aminotransferase (ALT), and lactate dehydrogenase (LDH), indicate metabolic stress, consistent with findings by Elarabany et al. (2017) and Enayati et al. (2013). These biochemical disruptions reflect the energetic costs associated with maintaining ionic homeostasis under saline conditions.

### Effects of ammonia nitrogen

Ammonia nitrogen concentrations exceeding 0.02 mg/L significantly affected survival, growth, and metabolic responses. Ammonia toxicity, indicated by elevated serum AST and ALT levels, points to liver damage and impaired protein metabolism, as corroborated by studies on ammonia toxicity in other aquaculture species (Chen et al. 2012). The daily measurement and adjustment of ammonia levels in this study ensured experimental uniformity, enhancing the reliability of these findings. At the molecular level, increased ammonia exposure triggered a downregulation of insulin-like growth factor I (IGF-I), indicative of suppressed growth pathways, and an upregulation of tumor necrosis factor-alpha (TNFα), reflecting heightened immune system activation. These findings build on earlier works (Dawood et al. 2020; El-Leithy et al. 2019) by linking physiological stress with specific gene expression changes, offering a mechanistic understanding of ammonia toxicity.

### Effects of stocking density

Stocking density exerted a comparatively moderate influence on performance metrics, with optimal growth observed at 15 fish per aquarium (15 fish/96 L). Higher stocking densities led to a modest reduction in growth, possibly due to increased competition and resource limitation. However, effective water quality management in this study mitigated the stress typically associated with overcrowding, as suggested by Bhujel (2014). These findings contrast with reports by El-Sayed and Kawanna (2008), who documented more pronounced effects of high-density conditions, emphasizing the importance of maintaining water quality to minimize stress.

### Broader implications for aquaculture

The study provides actionable recommendations for sustainable aquaculture practices. By identifying critical salinity and ammonia thresholds and optimal stocking densities, the research offers a framework for improving production efficiency while ensuring fish health. These findings are particularly relevant in the context of climate change and freshwater resource scarcity, which necessitate adaptive aquaculture strategies. Furthermore, the integration of molecular and biochemical analyses highlights the potential of using biomarkers, such as IGF-I and TNFα, for early detection of environmental stress. This approach could lead to the development of diagnostic tools for real-time health monitoring in aquaculture systems.

Despite the promising insights, this study has limitations. The controlled laboratory setting may not fully replicate the dynamic conditions of commercial aquaculture. Additionally, the focus on fingerlings limits the applicability of these findings to other life stages or long-term stress adaptations. Future research should address these gaps through field trials, multi-generational studies, and the development of stress-tolerant strains via selective breeding.

## Conclusion

This study advances the understanding of how salinity, ammonia nitrogen, and stocking density influence Nile tilapia fingerlings’ performance and health. By establishing specific tolerance thresholds and providing a holistic analysis of physiological, biochemical, and molecular responses, the research offers practical recommendations for improving tilapia farming practices.

The findings underscore the need for precise environmental control in aquaculture systems and demonstrate the utility of molecular biomarkers as tools for early stress detection. These insights contribute to the broader goal of sustainable intensification in aquaculture, addressing challenges associated with resource constraints and climate change.

Future research should focus on validating these findings in commercial-scale operations, exploring mitigation strategies for environmental stressors, and investigating long-term adaptability across life stages and generations. The development of genetically resilient strains presents a promising avenue for enhancing aquaculture sustainability and productivity.

In conclusion, this study provides a strong foundation for optimizing Nile tilapia farming practices, enhancing production efficiency, and minimizing environmental impacts. As aquaculture plays an increasingly critical role in global food security, these findings represent a significant step toward more sustainable and resilient food production systems.

## Data Availability

No datasets were generated or analysed during the current study.
